# Diagnostic uncertainty in a severely demented male patient: a case report

**DOI:** 10.1186/1757-1626-1-250

**Published:** 2008-10-19

**Authors:** Pantelis Maiovis, Evgenia Gavopoulou, Marina Eleftheriou, Ioanna Tsokanari, Magda Tsolaki

**Affiliations:** 1 Home Care Team for demented patients, Greek Association of Alzheimer's Disease and Related Disorders, Greece; 2 Assistant Professor of Neurology, Aristotle University of Thessaloniki, President of the Greek Association of Alzheimer's Disease and Related Disorders, Greece

## Abstract

**Introduction:**

Current trends in dementia research focus on early and accurate diagnosis. In clinical practice however, this is not always possible, as multiple underlying pathologies produce mixed dementia syndromes. Furthermore, patients with severe dementia are often underestimated.

**Case presentation:**

We present a case of a 71 year old Caucasian male with severe Alzheimer's Disease, bedridden and fully dependent in activities of everyday living, whose general cognitive function is almost intact. We emphasize on the diverse underlying pathologies contributing to this intriguing clinical presentation and to diagnostic uncertainty.

**Conclusion:**

Understanding the complexity of the dementia process in every patient using a multidimensional approach, contributes to more rational management strategies and finally to high quality care for patients and caregivers.

## Introduction

Dementia is a disabilitating syndrome resulting in progressive cognitive and functional decline as well as behavioral disorders. Given the severe burden of dementia worldwide, research is focusing on accurate and early diagnosis [1,2].

In clinical practice, however, accurate diagnosis following strict criteria is not usually the case, as multiple underlying pathophysiological mechanisms contribute to mixed dementia types [3]. Furthermore, concentrating on early diagnosis underestimates the importance of providing quality care for patients in severe stages [4]. Emerging trends suggest the need for a multidimensional and interdisciplinary approach to mild and especially severely demented patients [5], at home when appropriate [6]. Home care team of the Greek Association of Alzheimer's Disease and Related Disorders (consisting of a medical doctor, a dentist, a psychologist and a social worker) provides this kind of care.

We report a case of a patient with a diagnosis of severe 'Alzheimer's Disease' (AD), with almost intact cognitive abilities.

## Case presentation

A 71-year old Caucasian male, diagnosed with AD for the past 7 years, was referred because of severe mobility and behavioral problems. The patient, a former civil servant, with 16 years of education was in good general health 7 years before his referral to our team. At that time, he started to exhibit memory and executive function disorders, after experiencing severe psychological stress. He became increasingly irritable and started to repeat the same questions. He was depressed and suicidal most of the time; he once attempted to commit suicide. Aggressiveness, agitation, delusions and illusions developed later on. He was hospitalized for 5 months in a psychiatric clinic. After discharge, orientation disorders and confusion developed. He was prescribed with antidepressants, atypical antipsychotics and cholinesterase inhibitors. However, he noticed no improvement; rather, he suffered from drug adverse events. Since then, he has been repeatedly hospitalized for acute cholecystitis-pancreatitis, surgical treatment of benign prostate hyperplasia, and two strokes. Hypothyroidism was diagnosed and treated with thyrohormone per os. He developed pressure ulcers in the sacrococcygeal region, for which he had undergone surgical rehabilitation. Since August 2006, the patient is bedridden. In July 2007, the patient was referred to our team.

On examination, the patient was bedridden, cahectic, with pale conjunctivae. He was alert and sufficiently communicating but looked chronically ill, in no acute distress. On neurological examination, he was oriented in place and self but not in time and his speech was sometimes dysarthric. Examination of cranial nerves, sensory and cerebellar function revealed no abnormalities. Motor system examination revealed left hemiparesis, spasticity of the limbs, generalized muscular atrophy and weakness and increased tendon reflexes bilaterally. On oral evaluation, the patient had no teeth, consumed liquid and blended food and suffered from tongue and oral cavity mycosis.

Neuroimaging studies revealed frontal atrophy, white matter hyperintensities and a former ischemic infract in the right pons. Laboratory studies confirmed only anemia.

Neuropsychological examination yielded intriguing findings. The Mini Mental State Examination (MMSE) [7] was 24/30 suggesting that global cognitive function is satisfactory given his 7-year history with AD. The Geriatric Depression Scale (GDS) [8] was 9/15, confirming the clinical impression of depression.

Finally, on psychosocial evaluation, the family was in debt due to medical expenses, was socially isolated due to the stigma accompanying dementia and its coherence was fragile. The caregiver (patient's wife) was prematurely retired and suffered the physical and emotional burden of the 24-hour care of a bedridden patient.

## Conclusion

This case illustrates the complexity of the clinical presentation of dementia. The patient presented with executive, behavioral and emotional problems after a severe psychosocial stressor. Frontal lobe atrophy was dominating in neuroimaging exams. Vascular factors contributed to the present severe disability. Finally, thyroidal dysfunction may have added to the emotional instability and cognitive deficits.

In this case, diagnosis of Alzheimer's dementia is unlikely. Retrospective nature of the data collected makes it difficult either to reach an accurate diagnosis or to evaluate the quality of care provided to the patient, but we believe that the patient suffered from a frontal lobe dysfunction with depression, worsened by vascular and metabolic factors. The wrong diagnosis at the beginning as well as suboptimal care during the course of his illness may have caused the present severe disability.

The interdisciplinary approach followed by our team, led to better understanding of the disease course, ensuring a rational and problem-based management strategy and, ultimately, improving quality of life in the final stages of the disease [9]. Furthermore, referral of the patient's wife to special support and education groups run by the Greek Association of Alzheimer's Disease and Related Disorders, contributed to the reduction of the multi-dimensional burden of caring for a severely ill patient [10].

## Competing interests

The authors declare that they have no competing interests.

## Authors' contributions

PM, EG, ME and IT participated in the evaluation and care of the patient and his caregiver. MT coordinated and directed the work. PM involved in writing the manuscript. EG, ME and IT assisted in writing the manuscript. EG, ME, IT and MT reviewed the manuscript. All authors read and approved the final manuscript.

## Consent

Written informed consent was obtained from the patient's wife for publication of this case report. A copy of the written consent is available for review by the Editor-in-Chief of this journal.
